# Facilitatory effect of low-pulse repetition frequency ultrasound on release of extracellular vesicles from cultured myotubes

**DOI:** 10.1007/s10396-024-01429-9

**Published:** 2024-04-04

**Authors:** Xiaoqi Ma, Atomu Yamaguchi, Noriaki Maeshige, Kento Tanida, Mikiko Uemura, Fuwen Lu, Hiroyo Kondo, Hidemi Fujino

**Affiliations:** 1 Department of Rehabilitation Science, Kobe University Graduate School of Health Sciences, 10-2 Tomogaoka 7-chome, Suma-ku, Kobe, Hyogo 654-0142 Japan; 2 Shanghai Yangzhi Rehabilitation Hospital (Shanghai Sunshine Rehabilitation Center), Tongji University School of Medicine, 2209 Guangxing Rd., Songjiang District, Shanghai, 201619 China; 3 Faculty of Health and Nutrition, Shubun University, 72 Momo Higashiyashiki, Yamato-cho, Ichinomiya, Aichi 491-0932 Japan

**Keywords:** Pulsed ultrasound, Pulse repetition frequency, Extracellular vesicles (EVs), Intracellular calcium, Myotubes

## Abstract

**Purpose:**

Extracellular vesicles (EVs) serve as carriers of intracellular factors with therapeutic effects, including tissue regeneration and attenuation of inflammatory responses. The majority of EVs in vivo are derived from skeletal muscle, which is reported to have anti-inflammatory effects. While high-intensity pulsed ultrasound (US) irradiation has been shown to promote EV secretion from myotubes, the impact of pulse repetition frequency, a US parameter affecting pulse length, on EV release remains unclear. This study aimed to investigate the impact of pulse repetition frequency of US on the release of EVs from myotubes.

**Methods:**

C2C12 myoblasts were used in this study. After differentiation into C2C12 myotubes, US was performed for 5 min at an intensity of 3.0 W/cm^2^, duty cycle of 20%, acoustic frequency of 1 MHz, and different pulse repetition frequencies (100 Hz, 10 Hz, or 1 Hz). After 12 h, EVs and cells were collected for subsequent analyses.

**Results:**

US did not cause a reduction in cell viability across all US groups compared to the control. The concentration of EVs was significantly higher in all US groups compared to the control group. In particular, the highest increase was observed in the 1-Hz group on EV concentration as well as intracellular Ca^2+^ level.

**Conclusion:**

This study investigated the effect of three different pulse repetition frequencies of US on the release of EVs from cultured myotubes. It is concluded that a low-pulse repetition frequency of 1 Hz is the most effective for enhancing EV release from cultured myotubes with pulsed ultrasound.

**Supplementary Information:**

The online version contains supplementary material available at 10.1007/s10396-024-01429-9.

## Introduction

Pulsed ultrasound (US), a widely adopted therapeutic regimen of physical agents, offers noninvasive, painless, and favorable safety characteristics compared to other stimulation modalities, making it applicable to a broad range of patients [1]. With a rich history, it is increasingly used for angiogenesis, pain relief, tissue healing, and enhancing tissue properties [1–4]. Previous studies have demonstrated that cavitation is one of the underlying mechanisms of effects of US therapy [5, 6]. Furthermore, cavitation has been utilized to facilitate the delivery of both small and large molecules, including proteins and DNAs into cells [7, 8]. It has also been shown that US-induced transient membrane pores can enhance cellular calcium (Ca^2+^) uptake [9, 10], which is one of the initiators for the release of extracellular vesicles (EVs) by cells [11, 12]. Indeed, it has been shown that cavitation triggers exocytosis and promotes EV release from cells [13], and US itself can promote the release of EVs [14].

EVs, which are composed of a lipid bilayer membrane, serve as carriers of donor cell proteins, lipids, nucleic acids, mRNA, and microRNA, facilitating intercellular communication [15, 16]. They have been shown to regulate a diverse range of biological processes; promote tissue regeneration, cell proliferation, and differentiation; and attenuate inflammatory responses [17–20], emphasizing their critical role in cellular physiology and potential as targets for therapeutic intervention. Therefore, promoting release of EVs from skeletal muscle can exert various effects. Previous studies have highlighted that systemic exercise and hypoxic environments can promote EV release [21–23]. However, as systemic exercise training is not always feasible for elderly adults and patients, and oxidative stress due to hypoxic conditions can cause cellular damage, US therapy has been proposed as an alternative approach to enhance EV production.

In vivo, skeletal muscle is a significant source of EVs [18]. Skeletal muscle is the largest organ in the human body, accounting for approximately 40% of human body weight, and plays a vital role in metabolism and endocrine functions [24, 25]. Furthermore, skeletal myotube-derived EVs can prevent macrophage inflammatory responses [20]. Consequently, discovering a method for promoting EV release from skeletal muscle cells is essential. Notably, previous research has demonstrated that high-intensity US irradiation promotes EV secretion from cultured myotubes without cytotoxicity [14]. Meanwhile, the optimal pulse repetition frequency, a parameter that affects the form of US, has been poorly studied and is currently set empirically or fixed at 100 Hz [26]. By changing the pulse repetition frequency, the pulse duration and number of pulses can be adjusted without altering the duty cycle. Pulse duration, in particular, influences production of ultrasonic cavitation [27]. Since cavitation has been suggested to facilitate EV secretion [13], the pulse repetition frequency may influence EV release. Hence, this study aimed to investigate the effects of pulse repetition frequency of US on release of EVs from cultured myotubes.

## Materials and methods

### Cell culture

C2C12 myoblasts (ATCC) were sustained in Dulbecco's modified Eagle medium (DMEM) combined with 10% fetal bovine serum. Upon attaining nearly 90% confluence, the differentiation of myoblasts into myotubes was initiated by altering the medium to DMEM supplemented with 2% horse serum, which was replaced every 2–3 days over a period of 7 days. Upon completion of differentiation, the C2C12 myotubes were subjected to pulsed US irradiation.

### Ultrasound irradiation

**Fig. 1:**
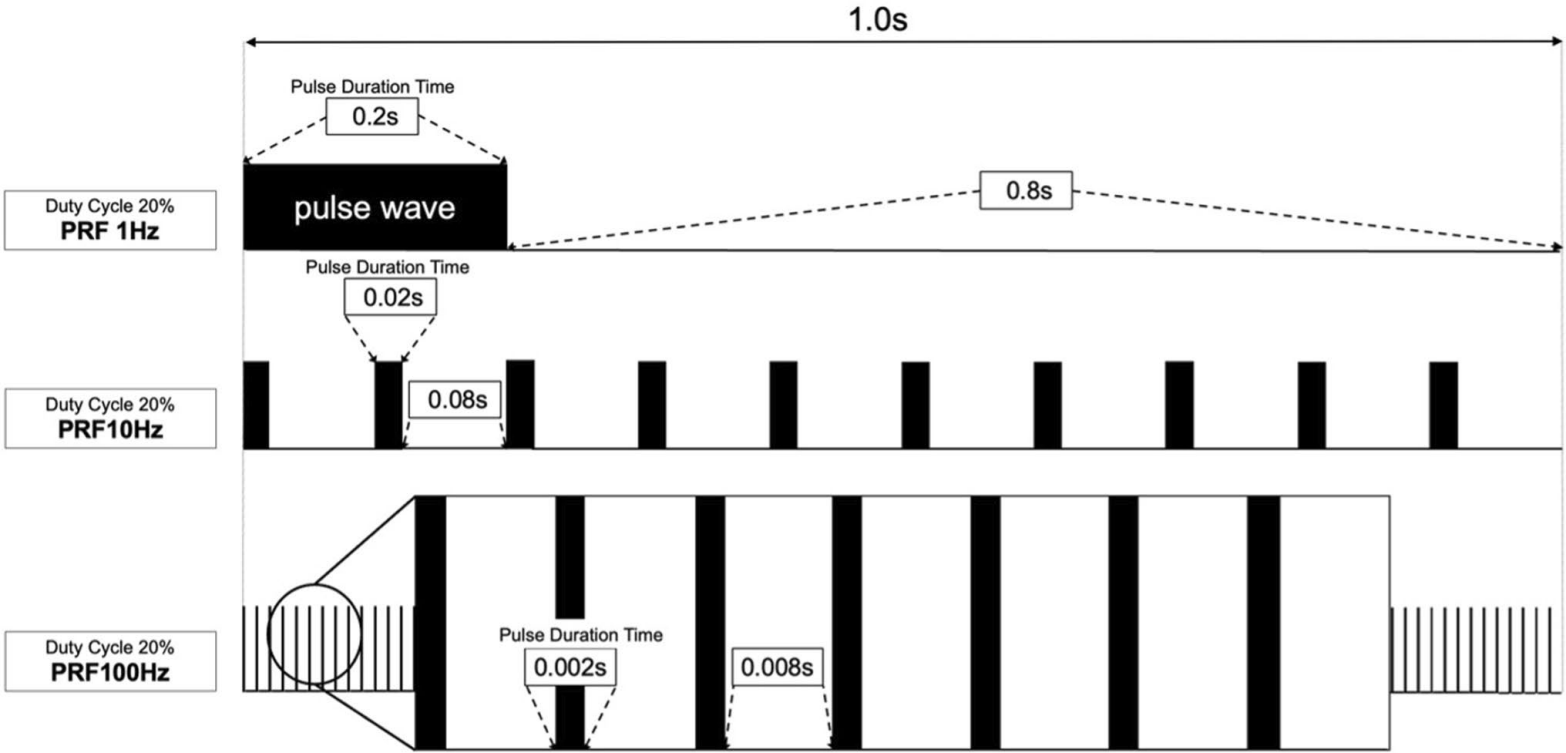
Illustration of ultrasound irradiation with different pulse repetition frequencies

**Fig. 2:**
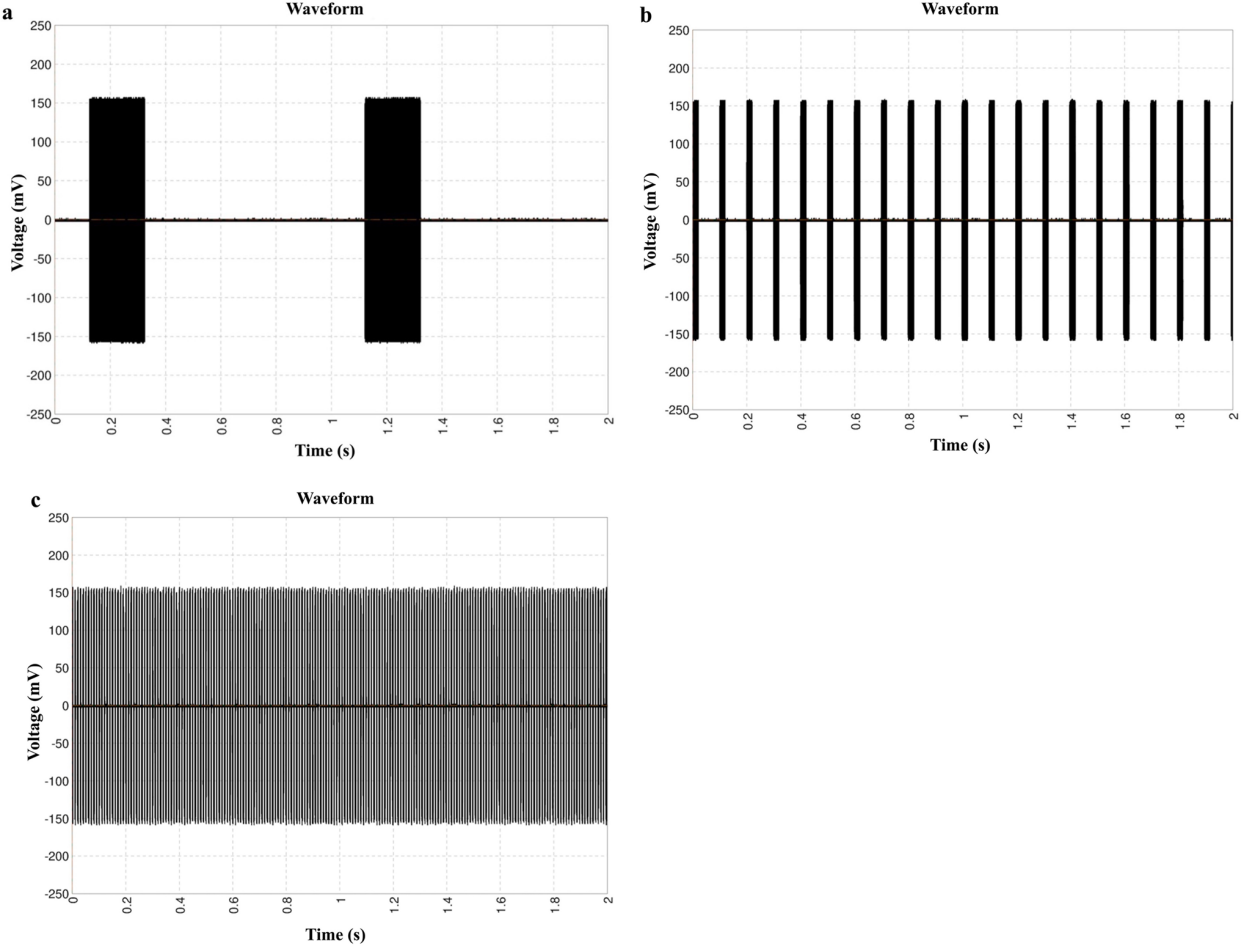
Actual waveforms of the different repetition frequencies of US used in this study. **a** the waveform of the repetition frequency of 1 Hz; **b** the waveform of the repetition frequency of 10 Hz; **c** the waveform of the repetition frequency of 100 Hz

After changing the culture medium to serum-free DMEM, US irradiation was conducted by positioning the probe of a customized medical US device (SZ-100 M; MINATO Medical Science Co., LTD, Japan), which allows adjustment to selectable repetition frequencies (100 Hz, 10 Hz, 1 Hz), under the bottom of the culture dish for 5 min (Supplemental File 1-Fig. 1). The amount of US energy transmitted through the dish bottom was approximately 82% of the total irradiated energy [8]. Coupling gel (< 1 mm) (Aquasonic 100 Ultrasound Transmission Gel; Parker Lab Inc., USA) was applied between the probe and the dish, and a piece of sterilized silicone was suspended in the culture media 2 mm above the cell monolayer [8, 14]. The US parameters were as follows: intensity of 3.0 W/cm^2^, duty cycle of 20%, acoustic frequency of 1 MHz, repetition frequencies of 1 Hz, 10 Hz, or 100 Hz (Fig. 1), and an effective radiation area of 7.6 cm^2^. Probe safety was confirmed with a beam nonuniformity ratio of 2.4 [8], calculated as the ratio of maximum to average US output. Additionally, the temperature of the culture media was monitored with a thermometer (TM-947SDJ; SATOTECH Co., LTD, Japan) to be below 37 °C during US irradiation (Supplemental File 1-Fig. 2). The temperatures between the US groups were also essentially the same.

Reference: Reference [13] has the same content with Reference [28] and [13] with [28]. Hence, to avoid repetition of references we have deleted the duplicate references [28, 30] and renumbered the subsequent references in ascending order. Please check if appropriateThank you for the revising.

### EV extraction and measurement

Twelve hours after US irradiation, the culture medium was collected and centrifuged at 1500×*g* for 10 min, followed by centrifugating the supernatant at 10,000×*g* for 30 min at 4 °C. Subsequently, a 0.22-µm filter was used to remove organelles and protein aggregates [28]. The supernatant was then ultracentrifuged at 100,000×*g* for 2 h at 4 °C, and the obtained pellet was suspended in 200 µl of D-PBS.

The particle concentration and size distribution of EVs were measured using a resistive pulse sensing device (qNano, Izon Science) with nanopores (NP200A, Izon Science). Calibration of size and concentration was accomplished using polystyrene beads (100 nm, Izon Science) with a concentration of 1.0 × 10^10^ beads/mL.

### Cell viability

The assessment of myotube viability was performed 12 h after US irradiation using MTT assay and Zombie Red™ staining. In the MTT assay, the cells were incubated for 3 h with 1.3 ml of MTT solution (10 ×); 3-(4,5-Dimethyl-2-thizolyl)-2,5-diphenyl-2 H-tetrazolium bromide (MTT; Wako Junyaku Co., Ltd., Japan) was dissolved in the culture medium at 5 mg/ml. After incubation for 3 h, dimethyl sulfoxide was added and the absorbance at 595 nm was measured (MTP-300; Kono Electric Co., Ltd., Japan). Cell viability was expressed as a percentage compared to the control.

In Zombie Red™ staining, the cells were washed with PBS twice and incubated for 15 min with Zombie Red™ Fiable Viability Kit (1:1000; BioLegend, CA, USA). Zombie Red™ is an amine-reactive fluorescent dye that is non-permeant to live cells but permeant to cells with a compromised plasma membrane. The cells were washed with PBS twice and incubated for 30 min at room temperature with 4% formaldehyde. Nuclei were counterstained with DAPI (1:1,000; Dojindo, Kumamoto, Japan) for 5 min. The stained cells were captured with a fluorescence microscope (BZ-X800; Keyence, Japan), and the mean fluorescence intensity was calculated.

### Intercellular Ca^2+^ concentration

The concentration of intracellular calcium was assayed immediately following US irradiation. Briefly, cells were collected using a cell scraper and then centrifuged at 13,000 rpm for 20 s to remove the supernatant. One microliter of hydrochloric acid was added to 200 µl of RIPA buffer (RIPA Lysis Buffer; SCB Co., Ltd., Japan) to maintain a pH of 2–3. Following 30-min incubation, samples were centrifuged at 10,000 rpm for 10 min to analyze the supernatant for calcium concentration as the low pH dissociates calcium, which is then collected as the supernatant. Calcium concentrations were analyzed by measuring absorbance at a sample wavelength of 690 nm and a reference wavelength of 770 nm using the Metalloassay Calcium Measurement LS (CPZ III) (Metallogenics Corporation).

### Statistical analysis

All values are expressed as means ± standard error of the mean (SEM). Statistical analysis was performed using Statistical 4 (OMS, Tokyo, Japan). For multiple comparisons, ANOVA (Tukey’s multiple comparison test as post-hoc) was used. p < 0.05 was considered statistically significant.

## Results

### Waveforms of different pulse repetition frequencies of US

In this study, waveforms of US irradiation were measured and are shown in Fig. 2. The measured waveforms of US irradiated with the pulse repetition frequencies of 1 Hz, 10 Hz, and 100 Hz over a period of 2 s are presented in Fig. 2a–c, respectively. They showed that the voltage of each pulse and the total irradiation time of the US was the same under different pulse repetition frequencies, but each pulse wave duration and the number of pulse waves were different under the different pulse repetition frequencies.

### Cell viability

**Fig. 3:**
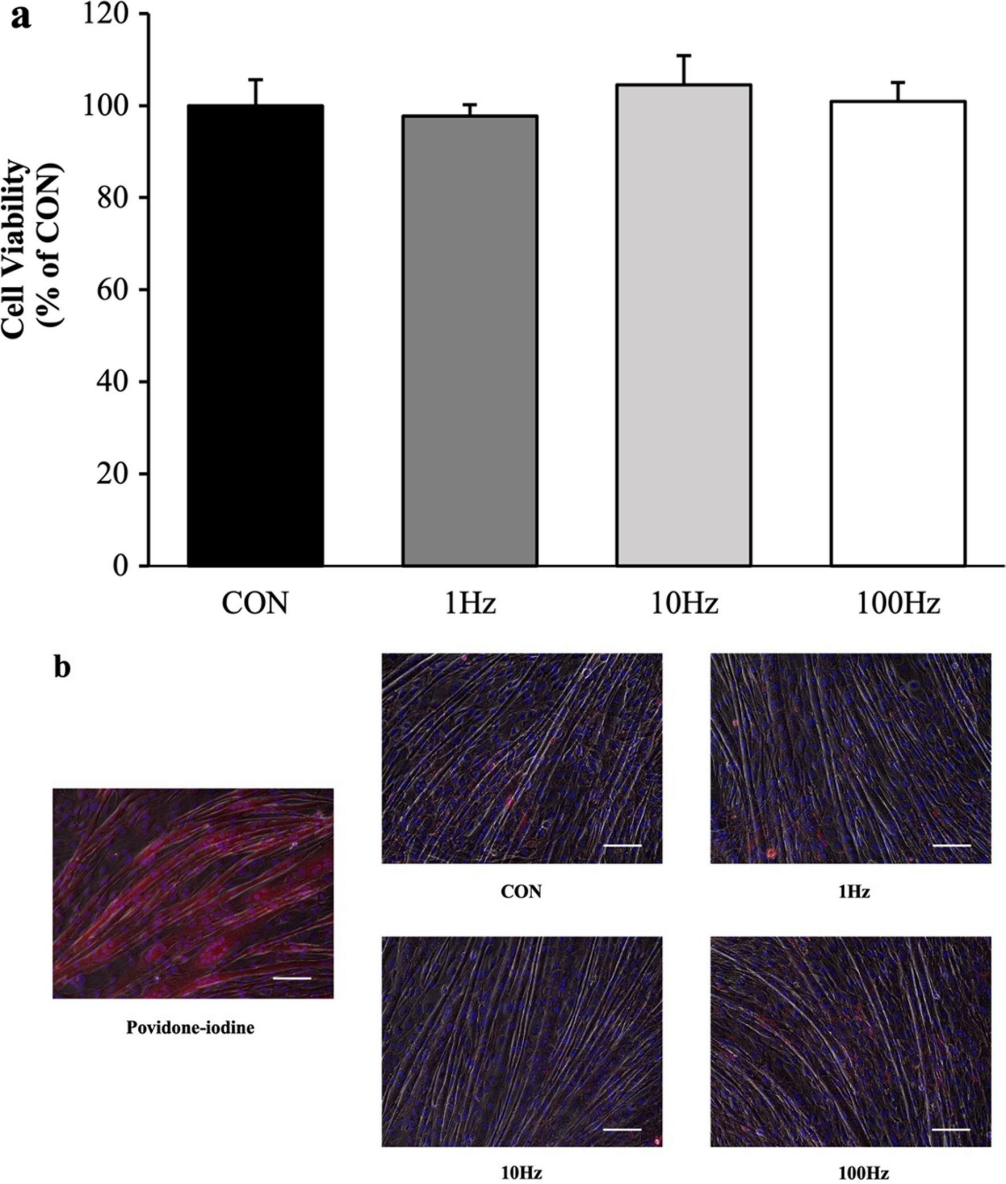
Cell viability. **a** MTT assay was performed at 12 h after ultrasound (US) irradiation for measuring cell viability, which is expressed as a percentage compared to the control. All values are represented as mean ± SEM (n = 4). **b** Zombie Red™ immunofluorescence staining (red) in myotubes at 12 h after US irradiation. After fixation, myotubes were counter-stained with DAPI (blue). Scale bar = 100 nm

As shown in Fig. 3a, the percentage of cell viability was not decreased by US irradiation. Also, as shown in Fig. 3b and Table 1, the cell viability of myotubes was measured using Zombie Red™ immunofluorescence staining to examine cytotoxicity of US irradiation on myotubes. While the cells treated with 1% povidone-iodine showed a significant decline in viability, US irradiation did not elicit a reduction in cell viability across all groups.

**Table 1:** Cell viability analysis using Zombie Red™ immunofluorescence staining. Mean fluorescence intensity of Zombie Red™ in myotubes is shown

Group	Mean fluorescence intensity (% of povidone-iodine)
Control	0.39 ± 0.12
100 Hz	0.38 ± 0.17
10 Hz	0.40 ± 0.08
1 Hz	0.41 ± 0.17

Values are presented as percentage of povidone-iodine and expressed as means ± SEM

### EV concentration and size distribution

**Fig. 4:**
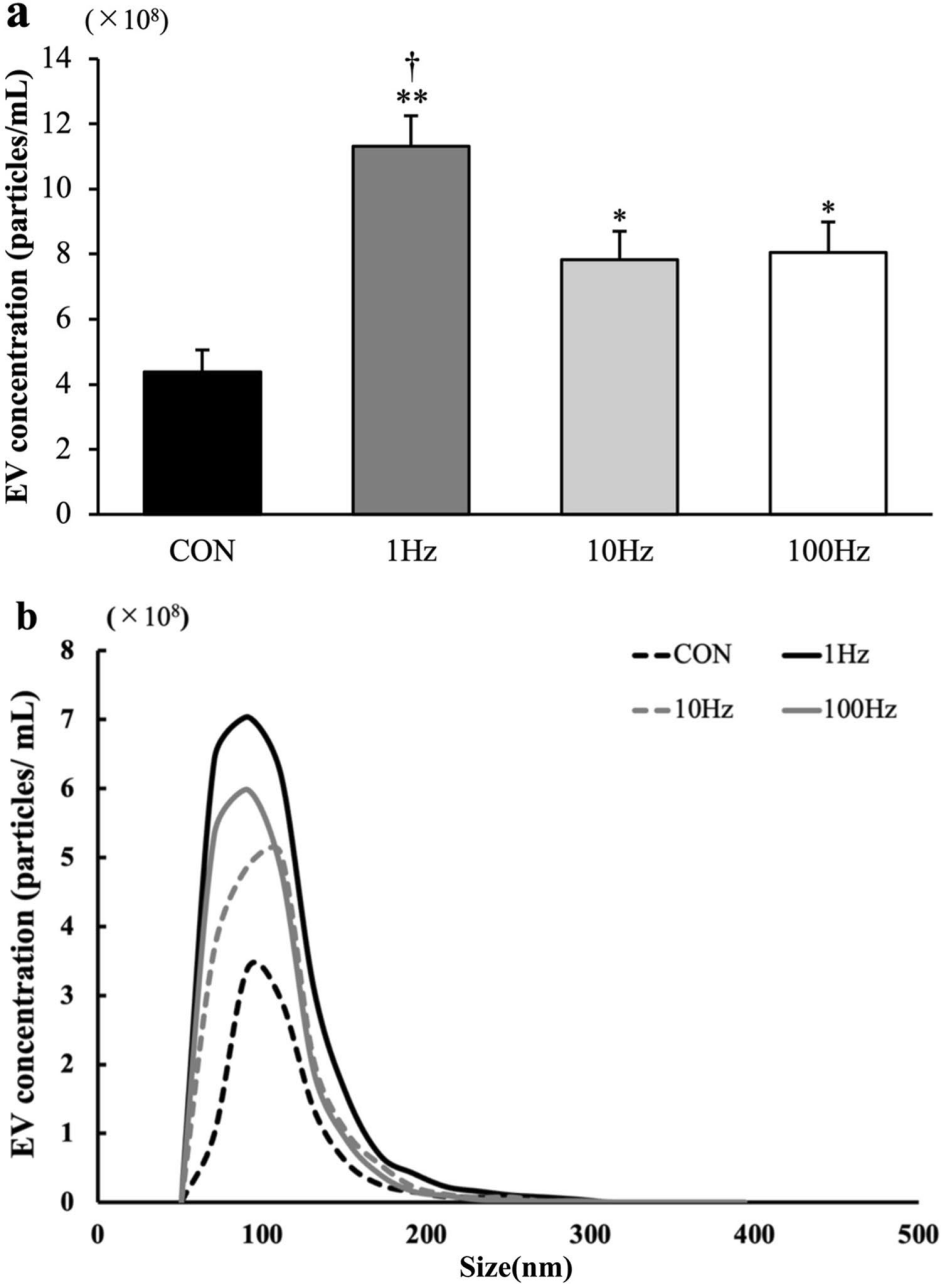
The concentration of extracellular vesicles (EVs). **a** The concentration of EVs from myotubes at 12 h after ultrasound (US) irradiation was measured using qNano. All values are represented as mean ± SEM.*p < .05 vs. control, **p < .01 vs. control, †p < .05 vs. 10 Hz (n = 9). **b** EV concentration and size distribution. The concentration and size distribution of EVs from myotubes at 12 h after US irradiation was measured (n = 9)

Isolated EVs were characterized using tunable resistive pulse sensing. In terms of EV concentration, all US groups showed a significant elevation relative to the control group. In particular, the highest increase was observed in the 1-Hz group, which was markedly greater than that in the 10-Hz group (Fig. 4a). Meanwhile, concerning the size of the extracted EVs, most of the particles were between 50 and 200 nm, the range of the EV size. There were no significant differences in EV size across all groups (Fig. 4b, Table 2).

**Table 2:** Extracellular vesicle (EV) distribution and size

Group	Mean (nm)	Mode (nm)
Control	113.1 ± 4.9	98.2 ± 4.6
100 Hz	109.0 ± 3.2	92.7 ± 4.0
10 Hz	112.3 ± 4.1	92.9 ± 5.4
1 Hz	114.8 ± 4.5	94.1 ± 4.1

The size distribution of EVs was measured. The mean and mode size of EVs in each group are shown in the table. All values are expressed as means ± SEM

### Intercellular Ca^2+^ concentration

**Fig. 5:**
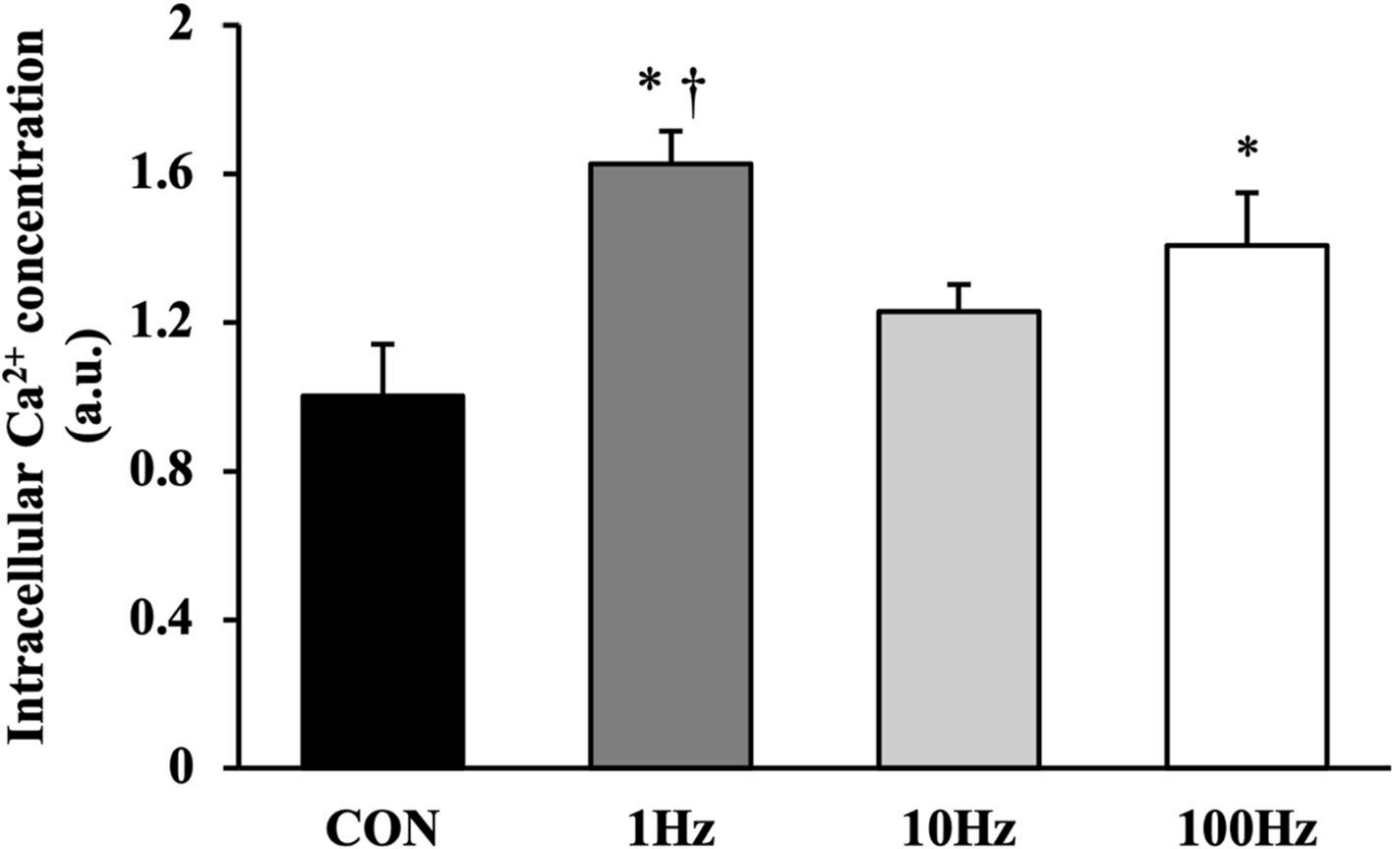
The concentration of intracellular Ca^2+^ was measured after ultrasound (US) irradiation. All values are represented as mean ± SEM.*p < .05 vs. control, †p < .05 vs. 10 Hz (n = 4)

To clarify the mechanism of US irradiation on the promotion of EV release, the intracellular Ca^2+^ concentration in myotubes after US irradiation was measured. The intracellular Ca^2+^ concentration after US irradiation was significantly elevated in the 100-Hz and 1-Hz groups as compared with the control group. Moreover, the 1-Hz group showed the highest concentration, which was significantly greater than that observed in the 10-Hz group, among the US groups (Fig. 5).

## Discussion

In this study, we investigated the promotion of release of EVs from cultured myotubes using different pulse repetition frequencies of US. The results of the MTT assay indicated that US irradiation at pulse repetition frequencies of 1 Hz, 10 Hz, and 100 Hz did not cause cell damage. This confirmed the non-injurious nature of US, which is consistent with a previous study in which cells were irradiated at a pulse repetition frequency of 100 Hz [14]. In the present investigation, the concentration of EVs was increased remarkably in all US groups, affirming the ability of US irradiation to stimulate the release of EVs. Additionally, the similarity in the size of the collected EVs among all groups suggested that the pulse repetition frequency of US did not influence the types of EVs. Of particular interest, the 1-Hz group exhibited a significantly higher concentration of EVs than the 10-Hz group, and had a tendency to increase the concentration of EVs by approximately 1.4 fold compared to the 100-Hz group. This result indicates that US irradiation at a pulse repetition frequency of 1 Hz was the most effective in promoting release of EVs from cultured myotubes in this study.

In previous studies, US irradiation was reported to promote uptake of Ca^2+^ from the extracellular space [13, 29]. Furthermore, an elevation in intracellular Ca^2+^ concentration was reported to encourage the release of exosomes, the most common type of EVs [30]. The present study confirmed that US irradiation elevated intracellular Ca^2+^ levels and the release of EVs, most notably at a pulse repetition frequency of 1 Hz. These findings suggest that the facilitation of Ca^2+^ uptake by US irradiation, in turn, increased the intracellular Ca^2+^ concentration and bolstered the excretion of EVs. Recently, the ESCRT complex, particularly ESCRT III, has been linked to exosome release via Ca^2+^ concentration increase [31, 32]. A further exploration focusing on these factors is needed.

Within the confines of our study, the greatest impact on intracellular Ca^2+^ levels was observed at a pulse repetition frequency of 1 Hz. Previous research has suggested that membrane displacement activates mechanosensory channels and, consequently, intracellular Ca^2+^ influx [33]. Additionally, it has been suggested that lower pulse repetition frequencies provoke greater cell membrane displacement [34]. Therefore, we hypothesized that the increase in cell membrane displacement at a repetition frequency of 1 Hz resulted in a higher intracellular Ca^2+^ concentration. Furthermore, US cavitation, which generates bubbles and jets that impact the cell membrane, has been identified as another factor contributing to cell membrane displacement [35, 36]. The jet stream generated by cavitation bubbles requires a certain degree of pulse duration, which is contingent on the acoustic frequency and intensity [37, 38]. In our study, the concentration of intracellular Ca^2+^ was significantly elevated at the pulse repetition frequency of 1 Hz, which showed the longest pulse duration. Therefore, we propose that the pulse repetition frequency of 1 Hz provides enough pulse duration to generate a jet stream of cavitation under US irradiation conditions. To confirm our hypotheses, further research regarding cavitation dynamics is warranted.

In the present study, we could not detect any difference between 100 and 10 Hz in terms of EV concentration and intracellular Ca^2+^ level. This means the length of the US pulse does not necessarily correlate to an increase in intracellular Ca^2+^ level. In addition, Ca^2+^ suppression experiments need to be conducted and factors related to mechanical stimulation analyzed in order to elucidate the proposed mechanism in the present study. Investigations on the effects of US on cavitation, membrane displacement, and mechanosensory channels will also be expected to clarify the biophysical action of low-pulse repetition frequency in future studies.

## Conclusion

This study revealed the effect of different pulse repetition frequencies of US on the facilitation of EV release from cultured myotubes. US irradiation with a pulse repetition frequency of 1 Hz significantly increased EV release and intracellular Ca^2+^ concentrations. Consequently, it is inferred that US irradiation with a pulse repetition frequency of 1 Hz is an effective way to facilitate EV release from skeletal muscles.

### Supplementary Information

Below is the link to the electronic supplementary material.

Supplementary file1 (DOCX 134 KB)

## Data Availability

The data sets used and analyzed during the current study are available from the corresponding author on reasonable request.
