# Supplementary material for: Facilitatory effect of low-pulse repetition frequency ultrasound on release of extracellular vesicles from cultured myotubes
Source: J Med Ultrason (2001). 2024 Apr 4;51(3):397–405. doi: 10.1007/s10396-024-01429-9 (PMC11272820; doi:10.1007/s10396-024-01429-9)
Supplement: Supplementary file 1 — Supplementary file1 (DOCX 134 KB) [file 10396_2024_1429_MOESM1_ESM.docx]

**Supplemental File 1**

**Supplemental File 1- 1:**


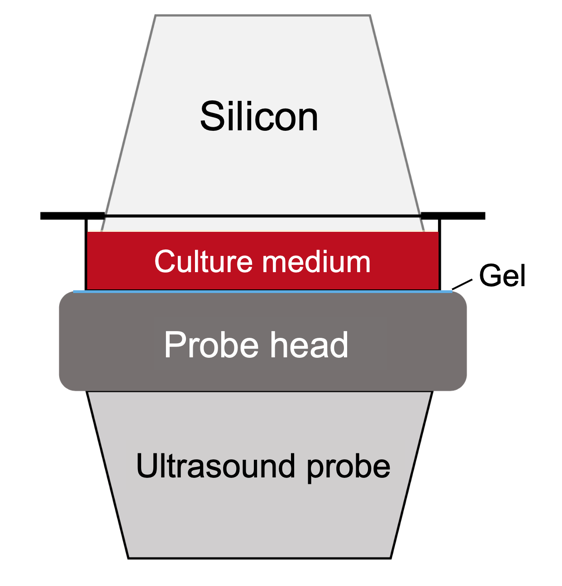


Supplemental 1- Fig.1 A cross-sectional figure of ultrasound irradiation system in vitro.

**Supplemental File 1- 2:** The culture medium temperature was below 37 degrees Celsius after US irradiation in all US groups.

*
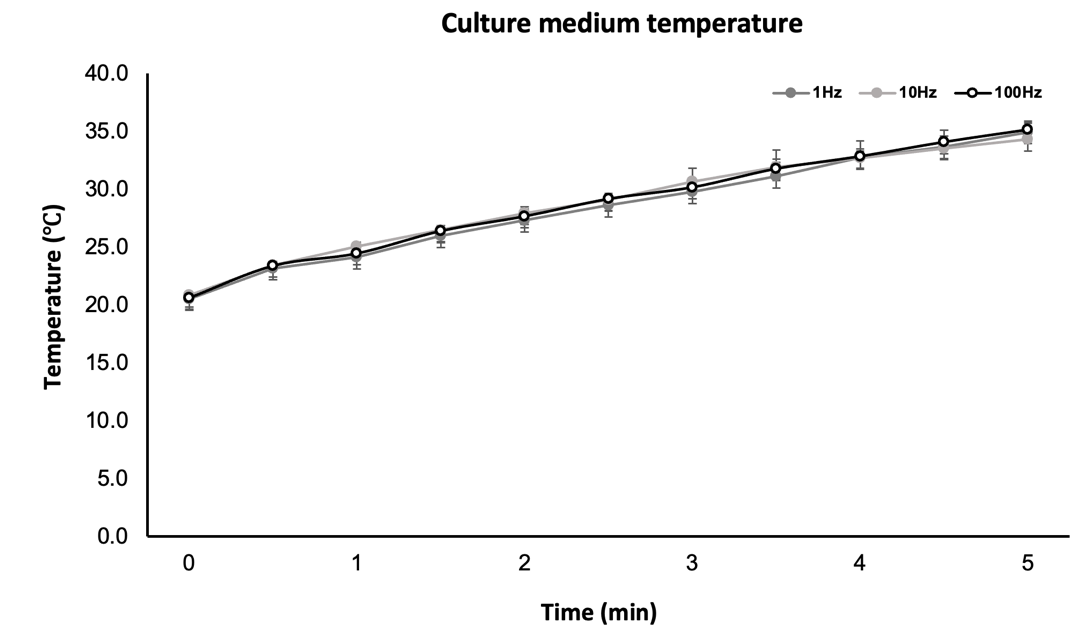
*

Supplemental 1- Fig.2 The temperature of the culture medium. During the US irradiation for 5min, the temperature was measured at the time of 0 min, 0.5 min, 1 min, 1.5 min, 2 min, 2.5 min, 3 min, 3.5 min, 4 min, 4.5 min, and 5 min (n = 3).
